# Type III chaperones & Co in bacterial plant pathogens: a set of specialized bodyguards mediating effector delivery

**DOI:** 10.3389/fpls.2013.00435

**Published:** 2013-11-22

**Authors:** David Lohou, Fabien Lonjon, Stéphane Genin, Fabienne Vailleau

**Affiliations:** ^1^Institut National de la Recherche Agronomique, UMR441, Laboratoire des Interactions Plantes-MicroorganismesCastanet-Tolosan, France; ^2^Centre National de la Recherche Scientifique, UMR2594, Laboratoire des Interactions Plantes-MicroorganismesCastanet-Tolosan, France; ^3^Institut National Polytechnique, École Nationale Supérieure Agronomique de Toulouse, Université de ToulouseCastanet-Tolosan, France

**Keywords:** bacterial plant pathogens, type III secretion system (T3SS), type III chaperones (T3Cs), type III secretion substrate specificity switch (T3S4), secretion of type III effectors (T3Es), control of secretion/translocation

## Abstract

Gram-negative plant pathogenic bacteria possess a type III secretion system (T3SS) to inject bacterial proteins, called type III effectors (T3Es), into host cells through a specialized syringe structure. T3Es are virulence factors that can suppress plant immunity but they can also conversely be recognized by the plant and trigger specific resistance mechanisms. The T3SS and injected T3Es play a central role in determining the outcome of a host-pathogen interaction. Still little is known in plant pathogens on the assembly of the T3SS and the regulatory mechanisms involved in the temporal control of its biosynthesis and T3E translocation. However, recent insights point out the role of several proteins as prime candidates in the role of regulators of the type III secretion (T3S) process. In this review we report on the most recent advances on the regulation of the T3S by focusing on protein players involved in secretion/translocation regulations, including type III chaperones (T3Cs), type III secretion substrate specificity switch (T3S4) proteins and other T3S orchestrators.

## INTRODUCTION

The type III secretion system (T3SS) is a major determinant of pathogenicity characterized in numerous Gram-negative animal and plant pathogenic bacteria (Cornelis and van Gijsegem, 2000; He et al., 2004; Tampakaki et al., 2010). It allows type III effector (T3E) delivery into the host cells thanks to a complex and ordered type III secretion (T3S) process (Büttner, 2012). T3Es are virulence factors that can suppress plant immunity or they can also conversely be recognized by the plant and trigger the so called effector-triggered immunity (Jones and Dangl, 2006; Feng and Zhou, 2012). This review is devoted to proteins that affect regulation of the secretion/translocation of T3Es in the two main groups of T3SS in plant pathogens, *i.e.*, Hrp1- (Hrp for hypersensitive response and pathogenicity) and Hrp2-T3SS phytopathogenic bacteria (see Tampakaki et al., 2010, and Büttner, 2012; for reviews on the genomic organization and architecture of T3SS). We give here an overview of the specialized type III chaperones (T3Cs) and of other T3S control proteins characterized in Hrp1 T3SS bacteria (*Pseudomonas syringae* and *Erwinia amylovora*), and in Hrp2 T3SS bacteria (*Xanthomonas *spp. and *Ralstonia solanacearum*).

## TYPE III CHAPERONES

**FIGURE 1:**
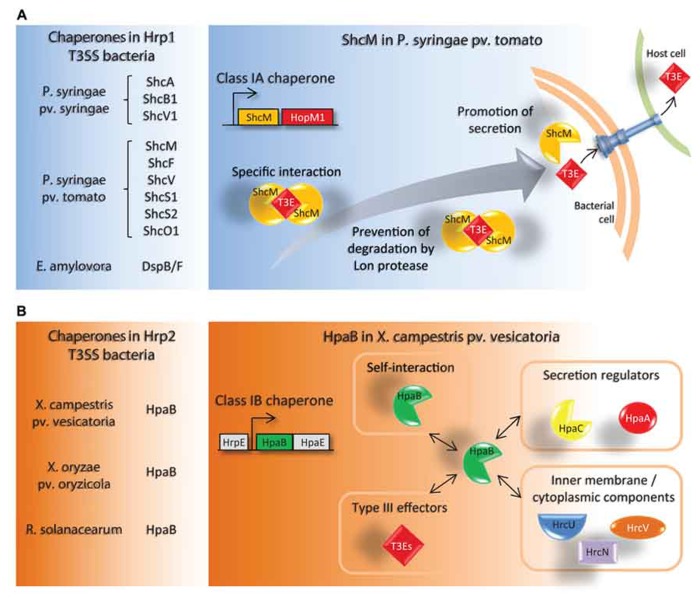
**Schematic representation of specific features of class IA and class IB T3Cs in plant pathogenic bacteria.** **(A)** List of known T3Cs in Hrp1 T3SS bacteria (left panel). All are class IA T3Cs. A model depicting the role of the T3C ShcM is shown as a representative example (right panel). ShcM is co-transcribed with its cognate T3E HopM1. ShcM specifically interacts with HopM1, prevents its degradation by Lon proteases in the bacterial cytoplasm and promotes its secretion/translocation *via* the T3SS. **(B)** List of known T3Cs in Hrp2 T3SS bacteria (left panel). All are class IB T3Cs. A schematic representation of known binding partners of HpaB in *X. campestris* pv. *vesicatoria* is shown (right panel). HpaB is encoded within the *hrp *cluster and without any T3E in the vicinity. HpaB interacts with itself and with multiple T3SS-related proteins, comprising the secretion regulators HpaC and HpaA (see also **Figure 2**), the inner membrane components HrcU and HrcV, the cytoplasmic ATPase HrcN and T3Es. HpaB is required for efficient secretion of T3Es.

Type III chaperones can be defined as helper proteins, mainly acting through direct interactions with T3Es, required for the delivery of effectors into the host cell. T3Cs are small (15–20 kDa), acidic, cytoplasmic proteins, and harbor a predicted α-helical secondary structure in their C-terminal part (Feldman and Cornelis, 2003; Parsot et al., 2003). T3Cs can be strictly or partially required to prevent cytoplasmic proteolysis and premature aggregation of T3Es, and/or maintain T3Es in a secretion competent status (Feldman and Cornelis, 2003; Parsot et al., 2003). T3Cs do not share amino acid sequence similarity, nevertheless, a classification has been proposed dividing them into three classes according to their cognate substrates. Class I is subdivided into classes IA and IB, corresponding to chaperones binding to one or several T3Es, respectively (Cornelis and van Gijsegem, 2000; Parsot et al., 2003). Class II chaperones that are specialized translocator-chaperones and class III chaperones, flagellar-associated T3SS chaperones, are up to now only described in animal pathogenic bacteria (see Büttner, 2012, as a review). We will focus on class I T3Cs as it is the main class described in phytopathogenic bacteria (**Figure 1**).

### CLASS IA T3C ARE MOSTLY INVOLVED IN THE SECRETION OF A SPECIFIC T3E

Class IA T3Cs were described in *P. syringae* pv. *syringae*, *P. syringae* pv. *tomato* and *E. amylovora*, all three pathogens harboring a Hrp1 T3SS (**Figure 1A**). The corresponding genes are located next to the cognate effector genes in the bacterial genome. These T3Cs were named Shc for Specific hop chaperones in *Pseudomonas* species as they help secretion of T3Es named Hops (*hrp*-dependent outer proteins), and to Dsp for disease specific protein, *i.e.*, chaperone or effector in *E. amylovora*. For all class IA T3Cs studied, a direct interaction T3C-cognate T3E has been demonstrated. To date, three Shc proteins have been characterized in *P. syringae* pv. *syringae*, including ShcA, which controls the secretion and translocation of HopA1 (van Dijk et al., 2002), and ShcB1 and ShcV1 that have been demonstrated to stabilize their cognate T3Es HopB1 and HopV1, respectively (Charity et al., 2003; Losada and Hutcheson, 2005). They prevent the T3E degradation mediated by the Lon protease, which exerts a negative regulatory effect on the T3S in *P. syringae* (Bretz et al., 2002). Three typical class IA T3Cs have been identified in *P. syringae* pv. *tomato*. ShcM plays several roles to promote efficient HopM1 translocation: it is required for the secretion and the translocation of HopM1 (Badel et al., 2003), but also protects HopM1 from Lon degradation, interacting with the effector through a chaperone binding domain (CBD) which is different from the Lon-targeting domain (Losada and Hutcheson, 2005; **Figure 1A**). ShcF is required for HopF stabilization but was shown dispensable for HopF secretion (Shan et al., 2004), whereas ShcV is required for HopV1 secretion (Wehling et al., 2004). Three other chaperones, SchS1, SchO1 and SchS2, harbor the typical features of T3Cs, but have atypical properties for class IA T3Cs as they could facilitate translocation of homologous T3Es (Kabisch et al., 2005). In another study, it was shown that SchS1, SchO1 and SchS2 were additionally able to bind to each other and to other’s cognate effectors (Guo et al., 2005). These three chaperones facilitate the secretion/translocation of their cognate T3E (HopS1, HopO1-1 and HopS2, respectively), and SchS1 and SchS2 can substitute ShcO1 to help HopO1-1 type III delivery (Guo et al., 2005). Other putative chaperones have been identified, located upstream a putative T3E target, but even if they show the physical properties of class IA T3Cs, their role still needs to be demonstrated. This is the case for the T3C-T3E pairs ShcN-HopN1 (Lopez-Solanilla et al., 2004) and ORF2-AvrE1 (Badel et al., 2006).

In *E. amylovora*, a putative T3C-T3E pair was characterized, the couple DspB/F-DspA/E (Gaudriault et al., 1997). DspB/F interacts with, stabilizes, and is important for DspA/E secretion (Gaudriault et al., 2002). Two studies identified the DspB/F binding sites in the N-terminal part of DspA/E, however with some differences in the regions of interactions (Triplett et al., 2009; Oh et al., 2010). This may indicate the presence of multiple CBD to help T3E translocation.

Class IA chaperones from phytopathogenic bacteria share common structural and functional features with class IA chaperones in animal pathogenic bacteria (He et al., 2004). The binding of both T3E and T3C partners is described in more details in the latter systems, notably through structural analysis of the T3C-T3E complex (Büttner et al., 2005; Lillington et al., 2011; Vujanac and Stebbins, 2013). Recently, co-crystal structure of the complex ShcA-HopA1 from *P. syringae* has also been obtained, both proteins sharing a fold and interacting through a conserved structural motif (called the β-motif) where HopA1 shows an extended non-globular conformation critical for the stability of the complex (Janjusevic et al., 2013). This β-motif is strongly conserved from animal to plant pathogens and was also described with the DspB/F chaperone of *E. amylovora* (Triplett et al., 2010), suggesting that it may be widely implicated in T3C-T3E complexes.

Another particular case of class IA chaperone is the HrpG protein which was described to act as a suppressor of a negative regulator of the T3SS (Wei et al., 2005). HrpG was characterized in *P. syringae *pv. *syringae* as a chaperone-like protein, as HrpG is structurally close to SicP, a class IA chaperone of *Salmonella typhimurium, *and harbors the characteristics of T3Cs (Wei et al., 2005). Wei et al. (2005) identified HrpG as an interactor of HrpV, a conserved component of the *P. syringae* T3SS, previously demonstrated to down-regulate the expression of the *hrp* gene cluster (Preston et al., 1998). Hence, the assembly of the HrpG–HrpV protein complex leads to the suppression of the *hrpV*-dependent negative regulation of the *hrp* gene cluster. More recently, Ortiz-Martin et al. (2010) studied HrpG in *P*. *syringae *pv. *phaseolicola* and highlighted for HrpG an additional HrpV-independent role in virulence.

### CLASS IB T3Cs ARE INVOLVED IN THE SECRETION OF SEVERAL T3Es

Genes which encode for class IB chaperones, binding several T3Es, are typically located within the *hrp* cluster. Class IB T3Cs were identified in several Hrp2 T3SS phytopathogenic bacteria, with one conserved protein named HpaB (for *hrp-*associated; **Figure 1B**). HpaB has been particularly well characterized in *X. campestris *pv. *vesicatoria*, as a T3C with a wide specificity of substrates. It was shown that HpaB could self-interact (Büttner et al., 2006) as well as with the inner membrane proteins HrcU and HrcV (Lorenz and Büttner, 2011; Hartmann and Büttner, 2013), with the ATPase HrcN (Lorenz and Büttner, 2009), with T3Es (AvrBs1 and AvrBs3; Büttner et al., 2004), and with other proteins involved in the T3S control (HpaC and HpaA; Büttner et al., 2004; Lorenz et al., 2008a; **Figure 1B**). HpaB promotes the secretion of many *Xanthomonas* T3Es (XopC, XopJ, AvrBst, AvrBs1, AvrBs3, and XopF1; Büttner et al., 2004, 2006). Like typical chaperones, HpaB is a small acidic protein, with *Yersinia*, *Shigella flexneri* or *Salmonella enteric*a T3Cs predicted similarities (Büttner et al., 2006), and with a rich content in leucine amino acids (Büttner et al., 2004), which may contribute to its ability to interact with various proteins. A HpaB homolog identified in *X. orizae *pv. *oryzicola* (Zou et al., 2006) was demonstrated to be involved in efficient translocation of 15 T3Es out of the 16 T3Es identified in this strain (Furutani et al., 2009). In *R. solanacearum*, HpaB was demonstrated to be required for efficient translocation of more than 66 T3Es (Mukaihara et al., 2004, 2010) but no data concerning putative interactions with T3Es is available. However, HpaB was shown to be dispensable for the translocation of harpin, pilin, and translocon proteins (Mukaihara et al., 2010). All these data support the view of a broader involvement of HpaB in T3S control rather than just a T3E escort function. This hypothesis is comforted by the *hrp*^-^ phenotype (no disease, no hypersensitive response, HR) of *hpaB* mutants, despite evidence for functional pili production in *X. campestris *pv. *vesicatoria* (Weber et al., 2005) or in *R. solanacearum* (Mukaihara et al., 2004). It also suggests that chaperone-mediated control for T3E delivery may be different according to the microorganism. The fact that specialized class II chaperones, which are responsible for the secretion of the translocon proteins in animal pathogenic bacteria, were not identified in phytopathogenic bacteria shows a noticeable difference compared to T3S regulators from phytopathogenic bacteria. Therefore, how secretion of translocon proteins is promoted in absence of class II T3Cs in plant pathogenic bacteria remains to be addressed.

## T3S4 PROTEINS

Based on the current knowledge of T3SS assembly, it is presumed that secretion of the Hrp pilus subunits must proceed before secretion of translocon proteins and effectors. This hierarchical process led to the classification of secreted proteins as “early” and “late” T3SS substrates. The involvement of proteins controlling T3S export process were first described as early as 1995 with the identification of the InvJ protein from *S. typhimurium* (Collazo et al., 1995). However, the link between functionally related proteins and the presence of a conserved T3S4 (type III secretion substrate specificity switch) domain was first described by Agrain et al. (2005a), with the characterization of the YscP protein from *Yersina* species. Members of the YscP family have little or no homology at the amino acid level, but are all proline-rich and harbor the so-called T3S4 domain in their C-terminal part. This structurally conserved domain is globular and composed of seven hydrophobic clusters of amino acids that define predicted β-strands and α-helices (Agrain et al., 2005a). *Yersinia* YscP protein has been particularly well characterized, highlighting a dual function: YscP is required to switch secretion from early to late substrates, including Yop (*Yersinia* outer proteins) T3Es (Edqvist et al., 2003; Agrain et al., 2005a) and controls the needle length, the nice molecular ruler model describing an attachment of the C-terminal part of YscP to the base of the secretion apparatus while the N-terminal part travels the inner channel of the growing needle. The needle is completed when its size corresponds to a fully stretched YscP protein (Journet et al., 2003; Agrain et al., 2005b). Since then, T3S4 proteins have been also intensively studied in other animal pathogenic bacteria, mainly in *Salmonella* (Kubori et al., 2000; Marlovits et al., 2006) and *Shigella* (Magdalena et al., 2002; Botteaux et al., 2008) species.

In plant pathogenic bacteria, the most studied T3S4 proteins are HpaC from *X. campestris* pv. *vesicatoria* (Büttner et al., 2006; Lorenz et al., 2008b) and HrpP from *P. syringae* (Morello and Collmer, 2009). Both proteins are important factors for bacterial pathogenicity. A *hpaC* mutant was shown to trigger reduced disease symptoms and reduced HR on susceptible and resistant pepper, respectively (Büttner et al., 2006). HrpP was found to be required for virulence on tomato and HR elicitation on *Nicotiana tabacum* (Morello and Collmer, 2009). Studies on HpaC and HrpP revealed that both proteins are involved at different levels in the regulation of T3S. In *X. campestris*, while the secretion of the Hrp pilin is not affected in a *hpaC* mutant, secretion of the translocon proteins (HrpF and XopA) and several T3Es (AvrBs3, XopC, XopJ and XopF1) is abolished (Büttner et al., 2006). On the other hand, in *P. syringae*, a *hrpP* mutant is severely impaired for pilin secretion but also for translocation of several T3Es such as AvrPto and therefore appears to behave almost like a T3SS-defective mutant (Ramos et al., 2007; Morello and Collmer, 2009). Quite surprisingly, HrpP itself is translocated into plant cells; however it was shown that the translocation of AvrPto is not dependent on the translocation of HrpP (Morello and Collmer, 2009).

**FIGURE 2:**
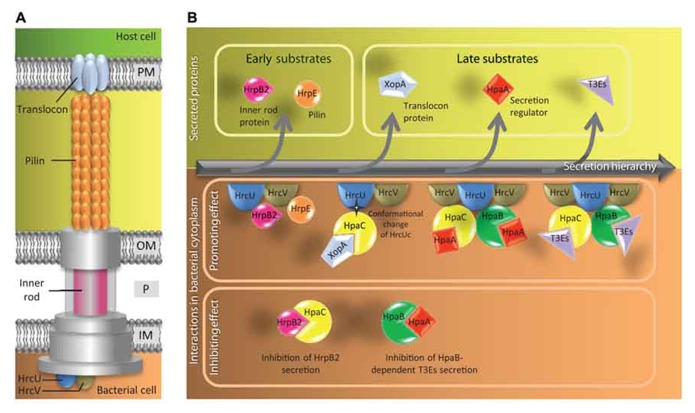
**Model of the control of type III secretion hierarchy in *X. campestris *pv. *vesicatoria*.** **(A)** Schematic representation of the T3SS highlighting components mentioned in **(B)**. The basal body of the T3SS spans the inner membrane (IM), the periplasm (P) and the outer membrane (OM) of the bacterium. The extracellular part of the T3SS comprises pilin subunits that form the pilus and translocon proteins that form a pore in the host plasma membrane (PM). The outer membrane/periplasmic inner rod is presumably the intracellular prolongation of the pilus. HrcU and HrcV are inner membrane components with a cytoplasmic C-terminal part. **(B)** Schematic view of the secreted proteins (upper part) and the protein interactions involved (lower part) during the hierarchical secretion process. HrcU and HrcV probably act as a docking platform for secretion. The first substrates that travel through the T3SS are the pilin HrpE and the inner rod protein HrpB2. Secretion of HrpB2 is required for secretion of HrpE. The switch of substrate specificity between early and late substrates (translocon proteins and T3Es) depends on a HpaC-mediated conformational change of the HrcU C-terminal domain (HrcUc). HpaC is a T3S4 protein that interacts with HrpB2 and the late substates. The switch between secretion of translocon proteins and T3Es requires the regulator HpaA. Secretion of HpaA releases the class IB chaperone HpaB. HpaB can bind to T3Es to promote their secretion.

To date, our understanding of T3S4 proteins is mostly based on the analysis of HpaC, a putative secretion switch factor regulating early (HrpB2 and HrpE pilin) and late (XopA translocator and T3Es) substrate secretion (**Figure 2**). HpaC appears to be a crossroad control protein with several interactors associated to different steps in the T3S process. Indeed, HpaC is able to self-interact and direct interactions with HpaC were identified with multiple partners including the inner membrane proteins HrcU and HrcV (Lorenz et al., 2008b; Hartmann and Büttner, 2013), the class IB chaperone HpaB (Büttner et al., 2006), the regulator HpaA (Lorenz et al., 2008a), the translocator XopA (Büttner et al., 2006), the ATPase HrcN (Lorenz and Büttner, 2009), and with T3Es (AvrBs3 and XopF1; Büttner et al., 2006; Lorenz et al., 2008b). HpaC was also shown to interact with HrpB2, an early T3S substrate required both for pilin subunit and translocators HrpF and XopA secretion (Rossier et al., 2000; Lorenz et al., 2008b; Hartmann et al., 2012). HrpB2 is part of the inner rod of the T3SS (Hartmann et al., 2012) and an interaction with the T3SS core component HrcU is necessary for its secretion (Lorenz and Büttner, 2011). HrpB2 is over-secreted in a *hpaC* mutant and in the same time, secretion of translocon proteins and T3Es is reduced (Büttner et al., 2006; Lorenz et al., 2008b). The HpaC-dependent substrate specificity switch requires the interaction between HpaC and the C-terminal domain of HrcU (HrcUc; Schulz and Büttner, 2011), that presumably induces a conformational change altering substrate specificity of the T3SS (Lorenz and Büttner, 2011). This change of conformation might be due to an autocatalytic cleavage of HrcU at a NPTH motif conserved between HrcU homologs in pathogenic bacteria. Mutations in the NPTH amino-acid motif of HrcU alter its interactions with both HpaC and HrpB2, suggesting that they may share the same binding site on HrcU (Lorenz and Büttner, 2011). Thus HpaC and HrpB2 could compete for their interaction with HrcU. It has been therefore proposed that HpaC could prevent efficient interaction between HrpB2 and HrcU and allow the access of the docking site of HrcU for secretion of translocon proteins and T3Es. But no interaction has been uncovered to date between HrcU and the T3SS substrates tested (XopA and XopF1; Lorenz et al., 2008b). However, it is noteworthy that the class IB HpaB was found to interact with HrcU (Lorenz and Büttner, 2011) and HrcV (Büttner et al., 2006; Hartmann and Büttner, 2013). It is conceivable that the docking of T3Es to HrcU and HrcV could be mediated by HpaB (**Figure 2**).

In *R. solanacearum*, the putative T3S4 domain protein HpaP is known to play a role in pathogenicity, since the corresponding mutant was reduced both for HR elicitation on tobacco and disease symptom production on tomato (van Gijsegem et al., 2002). In addition, HpaP was also required for an efficient secretion of the T3E PopA. Recent work characterized HpaP as a putative non-secreted T3S4 protein involved in the modulation of the secretion of early and late type III substrates, HrpY pilin and AvrA and PopP1 T3Es, respectively (Lohou et al., unpublished manuscript). As for HpaP homologs (Morello and Collmer, 2009; Schulz and Büttner, 2011), the T3S4 domain of *R. solanacearum* was demonstrated to be essential for HpaP’s role in virulence on tomato and *Arabidopsis thaliana* (Lohou et al., unpublished). Two other candidate T3S4 proteins studied in *X. oryzae* pv. *oryzae* and *X. oryzae* pv. *oryzicola*, both named HpaP, were shown to be important for pathogenicity on rice and for HR (Cho et al., 2008; Guo et al., 2012). In *X. oryzae* pv. *oryzae*, 16 T3Es have been found out to be T3SS substrates and all of them but one are dependent on HpaP for efficient translocation into tomato leaves (Furutani et al., 2009).

## ADDITIONAL T3S-ASSOCIATED REGULATORS: HELPERS, EFFECTORS OR BOTH?

Finally, there are reports in the literature of T3SS-associated proteins with an apparent dual role, being involved both in the control of the export process, being crucial for T3E secretion, but also being themselves translocated into the plant cell and described as virulence factors. The first example is *X. campestris *pv. *vesicatoria* HpaA, originally described as a *hrp*-associated protein, since the corresponding mutant was not able to trigger disease symptoms on susceptible pepper and tomato, but retained the ability to elicit a reduced HR on several resistant pepper and tomato lines (Huguet et al., 1998). Lorenz et al. (2008a) characterized HpaA as important for the secretion of different T3SS substrates (the pilin HrpE, the translocon proteins HrpF and XopA, and the T3Es AvrBs3, XopJ and XopC), as well as for the translocation of these T3Es. Two nuclear localization signals (NLS) were identified in HpaA, which was demonstrated to be secreted and translocated to the plant nucleus in a T3SS-dependent manner (Huguet et al., 1998; Lorenz et al., 2008a). Translocation of HpaA is HpaB-dependent, in agreement with the identified interaction between the two proteins (Alegria et al., 2004; Lorenz et al., 2008a).

The second example is HrpJ, a Hrp-associated protein found in *P. syringae* pathovars and *E. amylovora*. HrpJ was described as a T3S regulator required for *E. amylovora* pathogenicity as a *hrpJ* mutant displayed a reduced HR phenotype on *N. tabacum* and produced no disease symptoms on pear fruit (Nissinen et al., 2007). Bocsanczy et al. (2008) identified a direct interaction between HrpJ and the two harpins HrpN and HrpW. HrpJ was proposed to act as a chaperone to allow HrpN secretion, and both proteins are also required for DspA/E translocation, suggesting that HrpJ acts indirectly on the translocation of DspA/E by facilitating the secretion of HrpN (Nissinen et al., 2007; Bocsanczy et al., 2008). In *P. syringae* pv. *tomato*, the *hrpJ* mutant triggers a reduced HR in non-host tobacco plants and is also unable to provoke disease symptoms on *A. thaliana* (Fu et al., 2006). HrpJ was found to be required for the translocation of several T3Es (AvrB1, AvrRpt2, AvrPto1, AvrPtoB and HopB1; Fu et al., 2006). The secretion of the three harpins HrpZ1, HrpW1 and HopAK1, and of the translocon protein HrpK1 is HrpJ dependent (Fu et al., 2006; Crabill et al., 2012), whereas the pilus subunit HrpA1 is over-secreted in a *hrpJ* mutant (Crabill et al., 2012). These observations suggest that HrpJ controls the switch from secretion of the pilin to secretion of the harpins and of translocon proteins that are subsequently both involved in the interaction with the plant cell membrane. A *P. syringae* T3SS-deficient strain is able to grow better when inoculated on transgenic *A. thaliana* expressing HrpJ than on wild-type plants, and triggers two-fold less callose deposition (Crabill et al., 2012). This result suggested a role *in planta* for HrpJ as a T3E plant innate immunity suppressor (Crabill et al., 2012; Wei and Collmer, 2012) distinct from its known regulatory role during the biogenesis of the T3SS. The fact that HpaA is addressed to plant cell nucleus during infection could also be an indication that such proteins may have dual roles, being both involved in the control of the translocation process in the bacterium and in a more specific function as a T3E once inside the plant cell.

## CONCLUSIONS AND PERSPECTIVES

The current knowledge on T3Cs in plant pathogenic bacteria makes apparent a major difference between T3SS export control in Hrp groups 1 and 2: control of T3E export mainly proceeds in group 1 through class IA chaperones whereas group 2 seems to require class IB chaperones (**Figure 1**). It remains to be determined if this dichotomy reflects fundamental differences in T3SS export control mechanisms or just results from evolutionary divergence. T3S4 domain proteins appear as key players in the control of the secretion process. Future progress on the mechanistic T3S control will rely on more systematic identification of hetero-oligomeric protein complexes between the various T3SS control proteins identified, and on the evaluation of the different affinities between the interacting partners.

It is more than likely that T3S in bacterial plant pathogens is an ordered and sequential process, as demonstrated for T3SS of animal and human pathogens (Kubori and Nagai, 2011; Hicks and Galán, 2013). To date, observations supporting this view only relate on the assembly of the T3SS based on the discrimination between early and late T3SS substrates. However, there is still no direct evidence of a secretion hierarchy among T3Es in plant pathogens. This question of whether T3Es act in a coordinated spatial and temporal manner during host infection is particularly relevant for bacterial plant pathogens considering that they harbor large repertoires of translocated T3Es (Cunnac et al., 2009; Poueymiro and Genin, 2009). Future studies addressing this point should have important implications on T3E action inside host cells, as already demonstrated in other pathogens (Kubori and Galán, 2003). The recent development of microscopy-based assays opens exciting perspectives to provide a comprehensive description of dynamics of effector translocation and translocation temporal order (Mills et al., 2013).

## Conflict of Interest Statement

The authors declare that the research was conducted in the absence of any commercial or financial relationships that could be construed as a potential conflict of interest.
